# Supplementary material for: Bacillus subtilis YpeB holds SleB inactive, preventing cortex peptidoglycan degradation during spore dormancy
Source: mBio. 2026 Aug 7;17(9):e01316-26. doi: 10.1128/mbio.01316-26 (PMC13556211; doi:10.1128/mbio.01316-26)
Supplement: Supplementary file 1 [file mbio.01316-26-s0001.pdf]

***Bacillus subtilis* YpeB holds SleB inactive preventing cortex peptidoglycan degradation during spore dormancy**

**Yongqiang Gao, Jeremy D. Amon, Joshua C. Cofsky, David Z. Rudner**

**List of Supplementary Materials:**

**Plasmid constructions**
**Figures S1 - S8**
**Table S1 – S4**
**Supplemental References**

**Plasmid constructions**

**pYG34[*yvbJ::sleB (cat)(amp)*]** was constructed in a 2-way ligation with a PCR product containing *sleB* amplified with oCB120 and oYG121 using the gDNA of *B. subtilis 168* as template, and the plasmid pCB06 cut with EcoRI and BamHI. pCB06 is a is double crossover integration vector at the *yvbJ* locus with a *Cm* cassette.

**pYG530 [*MCS-proC His-SUMO-FLAG-ypeB (amp)*]** was constructed in a 2-way isothermal assembly reaction with a PCR product containing *ypeB* amplified with primers oY992 and oYG993 using gDNA of *B. subtilis* 168 as template, and the plasmid backbone derived from pLA73 (MCS-proC His-SUMO-FLAG-MCS) (1), which was restricted with BamHI and XhoI.

**pYG531 [*sleB His-SUMO-FLAG-ypeB (amp)*]** was constructed in a 2-way isothermal assembly reaction with a PCR product containing *sleB* amplified with primers oYG994 and oYG995 using gDNA of *B. subtilis* 168 as template, and the plasmid backbone was derived from pYG530 restricted with NcoI and NotI.

**pYG532 [*MCS-proC His-SUMO-FLAG-ypeB(Bc) (amp)*]** was constructed in a 2-way isothermal assembly reaction with a PCR product containing *ypeB(Bc)* amplified with primers oYG994-F and oYG995-R using gDNA of *B. cereus* as template, and the plasmid backbone was derived from pLA73 (MCS-proC His-SUMO-FLAG-MCS) (1), which was restricted with BamHI and XhoI.

**pYG533 [*sleB(Bc) His-SUMO-FLAG-ypeB(Bc) (amp)*]** was constructed in a 2-way isothermal assembly reaction with a PCR product containing *sleB(Bc)* amplified with primers oYG996 and oYG997 using gDNA of *B. cereus* as template, and the plasmid backbone was derived from pYG532 restricted with NcoI and NotI.

**pYG547 [*SP(dsbA)-sleB(Bc) His-SUMO-FLAG-ypeB(Bc) (amp)*]** was constructed in a 2-way isothermal assembly reaction with a PCR product containing *SP(dsbA)-sleB(Bc)*, in which the native signal peptide of *sleB(Bc)* was replaced with the signal peptide of *dsbA* in 3 steps: firstly the *sleB(Bc)*-1 was amplified with primers oYG997and oYG1015 using gDNA of *B. cereus* as template, and *sleB(Bc)-2* was derived from *sleB(Bc)-1* with primers oYG997 and oYG1016, and *SP(dsbA)-sleB(Bc)* was derived from *sleB(Bc)-2* with primers oYG997 and oYG1017. The plasmid backbone was derived from pYG532 with primers oYG190 and oYG1014.

**pYG548 [*SP(dsbA)-sleB His-SUMO-FLAG-ypeB (amp)*]** was constructed in a 2-way isothermal assembly reaction with a PCR product containing *SP(dsbA)-sleB*, in which the native signal peptide of *sleB* was replaced with the signal peptide of *dsbA* in 3 steps: firstly the *sleB-1* was amplified with primers oYG995 and oYG1018 using gDNA of *B. subtilis 168* as template, and *sleB-2* was derived from *sleB-1* with primers oYG995 and oYG1016, and *SP(dsbA)-sleB* was derived from *sleB-2* with primers oYG995 and oYG1017. The plasmid backbone was derived from pYG530 with primers oYG190 and oYG1014.

**pYG549 [*SP(pelB)-sleB(Bc) His-SUMO-FLAG-ypeB(Bc) (amp)*]** was constructed in a 2-way isothermal assembly reaction with a PCR product containing *SP(pelB)-sleB(Bc)*, in which the native signal peptide of *sleB(Bc)* was replaced with the signal peptide of *pelB* in 3 steps: firstly the *pelB-sleB(Bc)*-1 was amplified with primers oYG997and oYG1019 using gDNA of *B. cereus* as template, and *pelB-sleB(Bc)-2* was derived from *pelB-sleB(Bc)-1* with primers oYG997 and oYG1020, and *SP(pelB)-sleB(Bc)* was derived from *pelB-sleB(Bc)-2* with primers oYG997 and oYG1021. The plasmid backbone was derived from pYG532 with primers oYG190 and oYG1014.

**pYG550 [*SP(pelB)-sleB His-SUMO-FLAG-ypeB (amp)*]** was constructed in a 2-way isothermal assembly reaction with a PCR product containing *SP(pelB)-sleB*, in which the native signal peptide of *sleB* was replaced with the signal peptide of *pelB* in 3 steps: firstly the *pelB-sleB-1* was amplified with primers oYG995 and

oYG1022 using gDNA of *B. subtilis 168* as template, and *pelB-sleB-2* was derived from *pelB-sleB-1* with primers oYG995 and oYG1020, and *SP(pelB)-sleB* was derived from *pelB-sleB-2* with primers oYG995 and oYG1021. The plasmid backbone was derived from pYG530 with primers oYG190 and oYG1014.

**pYG566 [*SP(dsbA)-sleB-proC His-SUMO-FLAG-ypeB (amp)*]** was constructed in a 2-way isothermal assembly reaction with a PCR product containing *SP(dsbA)-sleB-proC* amplified with oYG1017 and oYG1051, using pYG548 as template, and the plasmid backbone was derived from pYG530 with primers oYG1050 and oYG190.

**pYG681 [*yvbJ::sleB-ypeB(spec)(amp)*]** was constructed in a 2-way ligation with a PCR product containing *sleB-ypeB* amplified with oYG105 and oYG106 using the gDNA of *B. subtilis 168* as template, and the plasmid pCB43 cut with EcoRI and BamHI. pCB43 is a is double crossover integration vector at the *yvbJ* locus with a *spec* cassette.

**pYG45 [*yvbJ::sleB-ypeB(cat)(amp)*]** was constructed in a 2-way ligation with a PCR product containing *sleB-ypeB* amplified with oYG105 and oYG106 using the gDNA of *B. subtilis 168* as template, and the plasmid pCB06 cut with EcoRI and BamHI. pCB06 is a is double crossover integration vector at the *yvbJ* locus with a *Cm* cassette.

**pYG638 [*yvbJ::sleB N190A-ypeB(cat)(amp)*]** was constructed by site-directed mutagenesis using primers oYG1187 and oYG1188, and pYG45 as template.

**pYG636 [*yvbJ::sleB N191A-ypeB(cat)(amp)*]** was constructed by site-directed mutagenesis using primers oYG1183 and oYG1184, and pYG45 as template.

**pYG689 [*yvbJ::sleB N190A N190A-ypeB(spec)(amp)*]** was constructed by site-directed mutagenesis using primers oYG1189 and oYG1190, and pYG681 as template.

**pYG640 [*yvbJ::sleB R194A Q198A-ypeB(cat)(amp)*]** was constructed by site-directed mutagenesis using primers oYG1191 and oYG1192, and pYG45 as template.

**pYG683 [*yvbJ::sleB F237A-ypeB(spec)(amp)*]** was constructed by site-directed mutagenesis using primers oYG1175 and oYG1176, and pYG681 as template.

**pYG684 [*yvbJ::sleB A181L-ypeB(spec)(amp)*]** was constructed by site-directed mutagenesis using primers oYG1195 and oYG1196, and pYG681 as template.

**pYG685 [*yvbJ::sleB A233L-ypeB(spec)(amp)*]** was constructed by site-directed mutagenesis using primers oYG1179 and oYG1180, and pYG681 as template.

**pYG686 [*yvbJ::sleB A181V-ypeB(spec)(amp)*]** was constructed by site-directed mutagenesis using primers oYG1193 and oYG1194, and pYG681 as template.

**pYG687 [*yvbJ::sleB A233V-ypeB(spec)(amp)*]** was constructed by site-directed mutagenesis using primers oYG1181 and oYG1182, and pYG681 as template.

**pYG688 [*yvbJ::sleB Y251A-ypeB(spec)(amp)*]** was constructed by site-directed mutagenesis using primers oYG1177 and oYG1178, and pYG681 as template.

**pYG703 [*yvbJ::sleB A233L-ypeB E101K(spec)(amp)*]** was constructed by site-directed mutagenesis using primers oYG1268 and oYG1269, and pYG685 as template.

**pYG705 [*yvbJ::sleB A181L-ypeB V87I(spec)(amp)*]** was constructed by site-directed mutagenesis using primers oYG1276 and oYG1277, and pYG684 as template.

**pYG706 [*yvbJ::sleB A181L-ypeB V185I(spec)(amp)*]** was constructed by site-directed mutagenesis using primers oYG1278 and oYG1279, and pYG684 as template.

**pYG707 [*yvbJ::sleB F237A-ypeB E101K(spec)(amp)*]** was constructed by site-directed mutagenesis using primers oYG1268 and oYG1269, and pYG683 as template.

**pYG708 [*yvbJ::sleB F237A-ypeB Q143R(spec)(amp)*]** was constructed by site-directed mutagenesis using primers oYG1270 and oYG1271, and pYG683 as template.

**pYG709 [*yvbJ::sleB F237A-ypeB V31A(spec)(amp)*]** was constructed by site-directed mutagenesis using primers oYG1272 and oYG1273, and pYG683 as template.

**pYG710 [*yvbJ::sleB F237A-ypeB H84R(spec)(amp)*]** was constructed by site-directed mutagenesis using primers oYG1274 and oYG1275, and pYG683 as template.

**pYG711 [*yvbJ::sleB F237A-ypeB V87I(spec)(amp)*]** was constructed by site-directed mutagenesis using primers oYG1276 and oYG1277, and pYG683 as template.

**pYG712 [*yvbJ::sleB F237A-ypeB V185I(spec)(amp)*]** was constructed by site-directed mutagenesis using primers oYG1278 and oYG1279, and pYG683 as template.

**pYG535 [*yhdG::sleB-ypeB(spec)(amp)*]** was constructed in a 2-way ligation with a PCR product containing *sleB-ypeB* amplified with oYG105 and oYG106 using the gDNA of *B. subtilis 168* as template, and the plasmid pCB33 cut with EcoRI and BamHI. pCB33 is a is double crossover integration vector at the *yhdG* locus with a *spec* cassette.

**pYG731 [*yhdG::sleB-ypeB H44A(spec)(amp)*]** was constructed by site-directed mutagenesis using primers oYG1319 and oYG1320, and pYG535 as template.

**pYG732 [*yhdG::sleB-ypeB E45A(spec)(amp)*]** was constructed by site-directed mutagenesis using primers oYG1321 and oYG1322, and pYG535 as template.

**pYG553 [*yhdG::sleB-ypeB H44A E45A(spec)(amp)*]** was constructed by site-directed mutagenesis using primers oYG1026 and oYG1027, and pYG535 as template.

**pYG556 [*yhdG::sleB-ypeB R147E(spec)(amp)*]** was constructed by site-directed mutagenesis using primers oYG1032 and oYG1033, and pYG535 as template.

**pYG757 [*yhdG::sleB-ypeB M154A(spec)(amp)*]** was constructed by site-directed mutagenesis using primers oYG1360 and oYG1361, and pYG535 as template.

**pYG758 [*yhdG::sleB-ypeB N157A(spec)(amp)*]** was constructed by site-directed mutagenesis using primers oYG1362 and oYG1363, and pYG535 as template.

**pYG557 [*yhdG::sleB-ypeB M154A N157A(spec)(amp)*]** was constructed by site-directed mutagenesis using primers oYG1034 and oYG1035, and pYG535 as template.

**pYG752 [*yhdG::sleB-ypeB Y280A(spec)(amp)*]** was constructed by site-directed mutagenesis using primers oYG1350 and oYG1351, and pYG535 as template.

**pYG753 [*yhdG::sleB-ypeB N284A(spec)(amp)*]** was constructed by site-directed mutagenesis using primers oYG1352 and oYG1353, and pYG535 as template.

**pYG559 [*yhdG::sleB-ypeB Y280A N284A(spec)(amp)*]** was constructed by site-directed mutagenesis using primers oYG1038 and oYG1039, and pYG535 as template.

**pYG736 [*yhdG::sleB-ypeB Y280A I282A N284A(spec)(amp)*]** was constructed by site-directed mutagenesis using primers oYG1329 and oYG1330, and pYG559 as template.

**pYG733 [*yhdG::sleB-ypeB I282A(spec)(amp)*]** was constructed by site-directed mutagenesis using primers oYG1323 and oYG1324, and pYG535 as template.

**pYG734 [*yhdG::sleB-ypeB T249A(spec)(amp)*]** was constructed by site-directed mutagenesis using primers oYG1325 and oYG1326, and pYG535 as template.

**pYG751 [*yhdG::sleB-ypeB K210A(spec)(amp)*]** was constructed by site-directed mutagenesis using primers oYG1335 and oYG1336, and pYG535 as template.

**pYG742 [*yhdG::sleB-ypeB K210A T249A(spec)(amp)*]** was constructed by site-directed mutagenesis using primers oYG1325 and oYG1326, and pYG751 as template.

**pYG735 [*yhdG::sleB-ypeB Y323A(spec)(amp)*]** was constructed by site-directed mutagenesis using primers oYG1327 and oYG1328, and pYG535 as template.

**pYG739 [*yhdG::sleB-ypeB I446A(spec)(amp)*]** was constructed by site-directed mutagenesis using primers oYG1331 and oYG1332, and pYG535 as template.

**pYG740 [*yhdG::sleB-ypeB Y323A I446A(spec)(amp)*]** was constructed by site-directed mutagenesis using primers oYG1331 and oYG1332, and pYG735 as template.

**pYG759 [*yhdG::sleB-ypeB T93A(spec)(amp)*]** was constructed by site-directed mutagenesis using primers oYG1364 and oYG1365, and pYG535 as template.

**pYG760 [*yhdG::sleB-ypeB L94A(spec)(amp)*]** was constructed by site-directed mutagenesis using primers oYG1366 and oYG1367, and pYG535 as template.

**pYG741 [*yhdG::sleB-ypeB T93A L94A(spec)(amp)*]** was constructed by site-directed mutagenesis using primers oYG1333 and oYG1334, and pYG535 as template.

**pYG754 [*yhdG::sleB-ypeB E444A(spec)(amp)*]** was constructed by site-directed mutagenesis using primers oYG1354 and oYG1355, and pYG535 as template.

**pYG755 [*yhdG::sleB-ypeB E444R(spec)(amp)*]** was constructed by site-directed mutagenesis using primers oYG1356 and oYG1357, and pYG535 as template.

**pYG767 [*yhdG::$P_{sleB}$-(HindIII-XhoI)-ypeB R147E (amp)*]** was constructed in a 2-way isothermal assembly reaction with a PCR product containing *$P_{sleB}$-(HindIII-XhoI)* amplified with oCB63 and oYG999, using pYG556 as template, and the plasmid backbone was also derived from pYG556 with primers oYG998 and oYG1002.

**pYG768 [*yhdG::$P_{sleB}$-(HindIII-XhoI)-ypeB E444R (amp)*]** was constructed in a 2-way isothermal assembly reaction with a PCR product containing *$P_{sleB}$-(HindIII-XhoI)* amplified with oCB63 and oYG999, using pYG755 as template, and the plasmid backbone was also derived from pYG755 with primers oYG998 and oYG1002.

**pYG779 [*yhdG::sleB N267I-ypeB R147E(spec)(amp)*]** was constructed by site-directed mutagenesis using primers oYG1407 and oYG1408, and pYG556 as template.

**pYG780 [*yhdG::sleB D192V-ypeB E444R(spec)(amp)*]** was constructed by site-directed mutagenesis using primers oYG1409 and oYG1410, and pYG755 as template.

**pYG783 [*yhdG::sleB N267I-ypeB E45K(spec)(amp)*]** was constructed by site-directed mutagenesis using primers oYG1407 and oYG1408, and pYG554 as template.

**pYG784 [*yhdG::sleB D192V-ypeB E45K(spec)(amp)*]** was constructed by site-directed mutagenesis using primers oYG1409 and oYG1410, and pYG554 as template.

**pYG796 [*yhdG::sleB D192V-ypeB M154A N157A(spec)(amp)*]** was constructed by site-directed mutagenesis using primers oYG1409 and oYG1410, and pYG557 as template.

**pYG799 [*yhdG::sleB N267I-ypeB M154A N157A(spec)(amp)*]** was constructed by site-directed mutagenesis using primers oYG1407 and oYG1408, and pYG557 as template.

**pYG802 [*yhdG::sleB D192V-ypeB(spec)(amp)*]** was constructed by site-directed mutagenesis using primers oYG1409 and oYG1410, and pYG535 as template.

**pYG803 [*yhdG::sleB N267I-ypeB(spec)(amp)*]** was constructed by site-directed mutagenesis using primers oYG1407 and oYG1408, and pYG535 as template.

All plasmids were confirmed by Sanger sequencing.

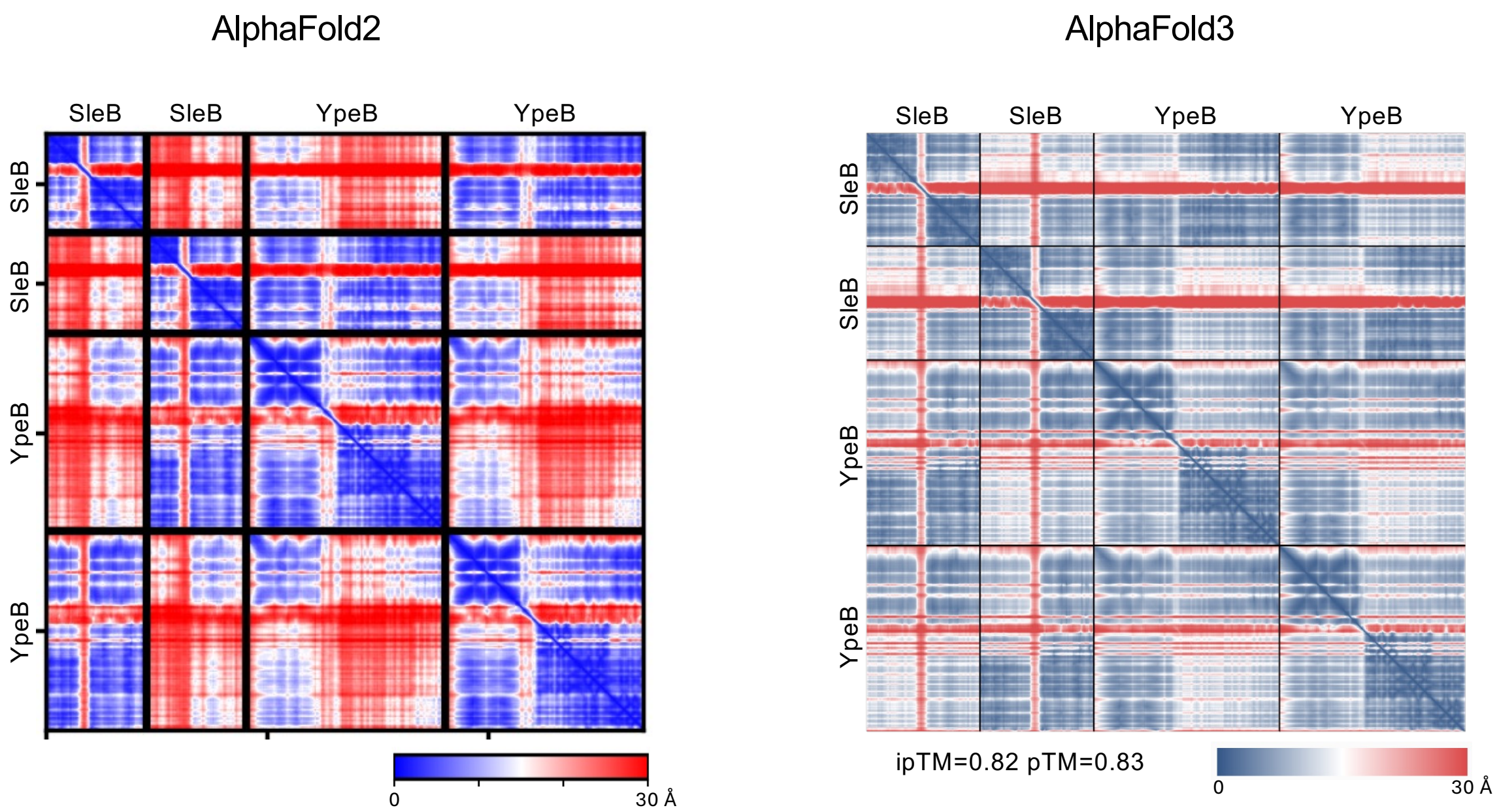


**Figure S1. Predicted alignment error (pAE) plots for SleB$_2$-YpeB$_2$ AlphaFold-Multimer predictions.** Plots of the predicted alignment error (pAE) in Å of all residues against all residues for the top-ranked AlphaFold2 and AlphaFold3 models for the SleB$_2$-YpeB$_2$ tetramer. Low error (blue) corresponds to well-defined relative domain positions. The interface predicted template modeling score (ipTM) and the template modeling score (pTM) for the AlphaFold3 model are shown below the plot.

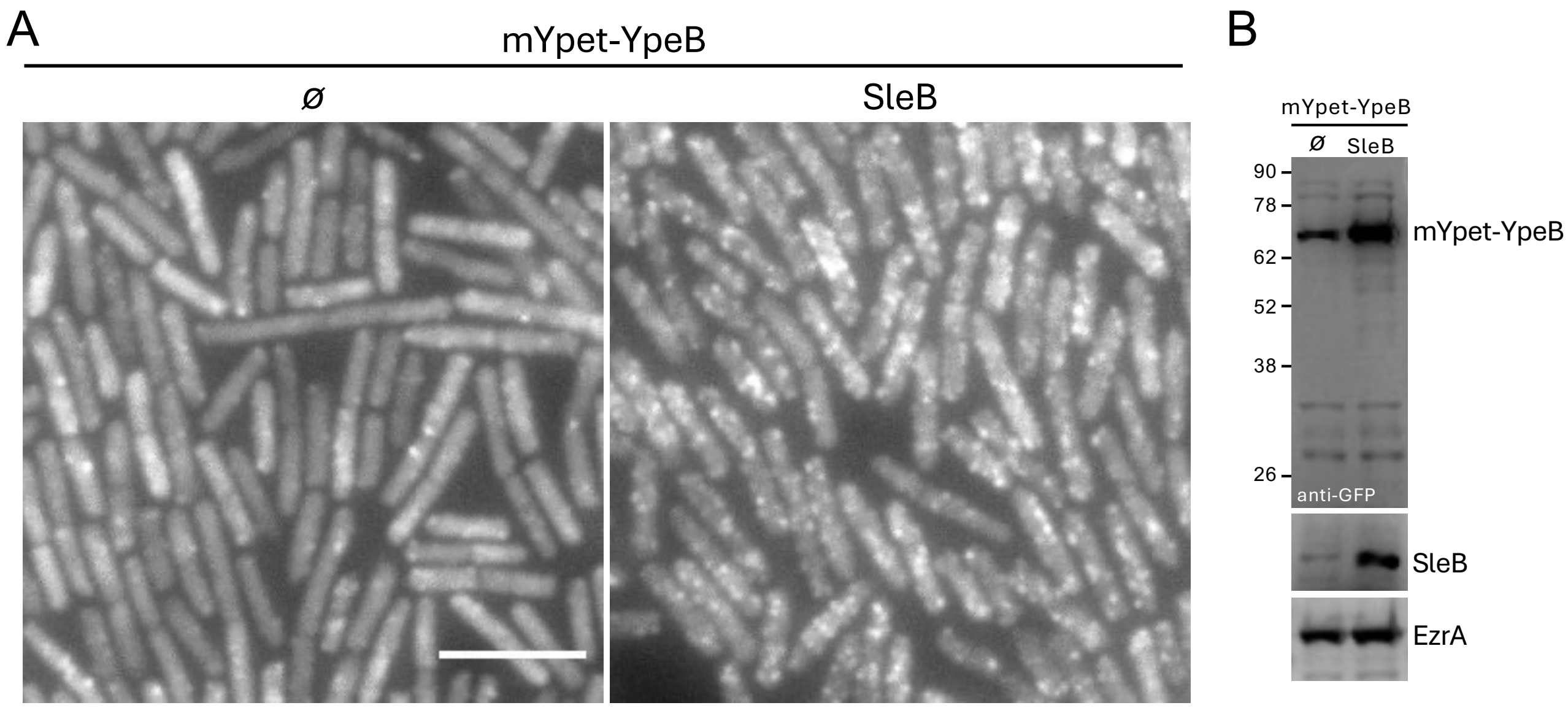


**Figure S2. SleB expression stabilizes YpeB and promotes mYpet-YpeB fluorescent foci when expressed during growth. (A)** Representative fluorescence images of exponentially growing *B. subtilis* cells expressing mYpet-YpeB in the absence (ø) or presence of SleB. The mYpet-YpeB fusion was expressed under the control of a xylose-regulated promoter ($P_{xylA}$) with 3.3 mM xylose, and SleB was expressed under the control of an IPTG-regulated promoter ($P_{hyperspank}$) with 1 mM IPTG. Scale bar indicates 5 µm. **(B)** Representative immunoblot of lysates from the strains in (A). mYpet-YpeB and SleB were detected with anti-GFP and anti-SleB antibodies. EzrA controls for loading. Molecular weight standards (in kDa) are shown on the left.

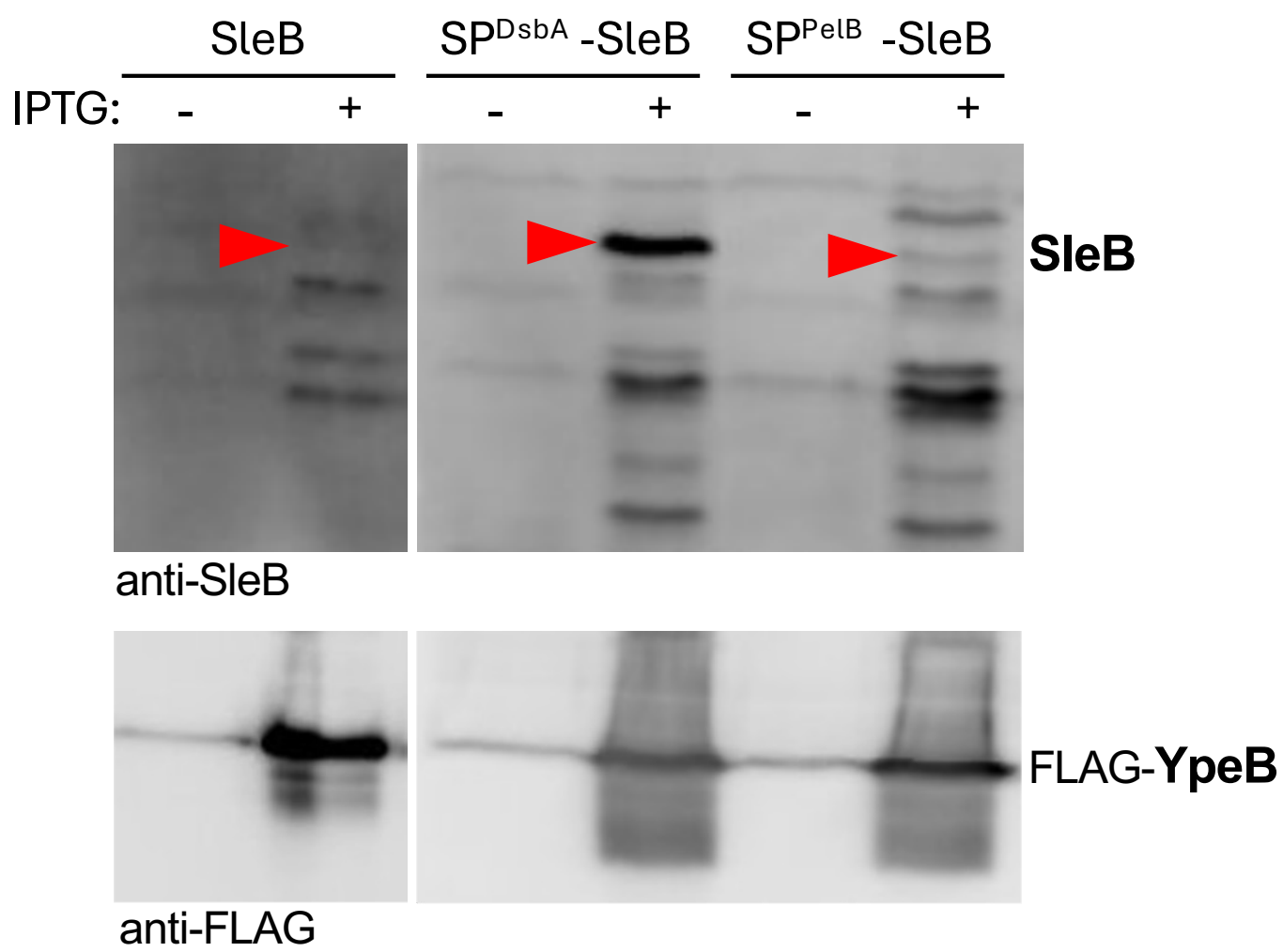


**Figure S3. *E. coli* expression of SleB is enhance when its native signal peptide is replaced with *E. coli*'s DsbA signal peptide.** Representative immunoblots of SleB and FLAG-YpeB co-expressed in *E. coli* with 0.5 mM IPTG. Cells expressing wild-type *sleB* and *sleB* variants with the signal peptide (SP) from *E. coli* DsbA and from *Erwinia carotovora* PelB are shown. The band corresponding to SleB is indicated (red caret).

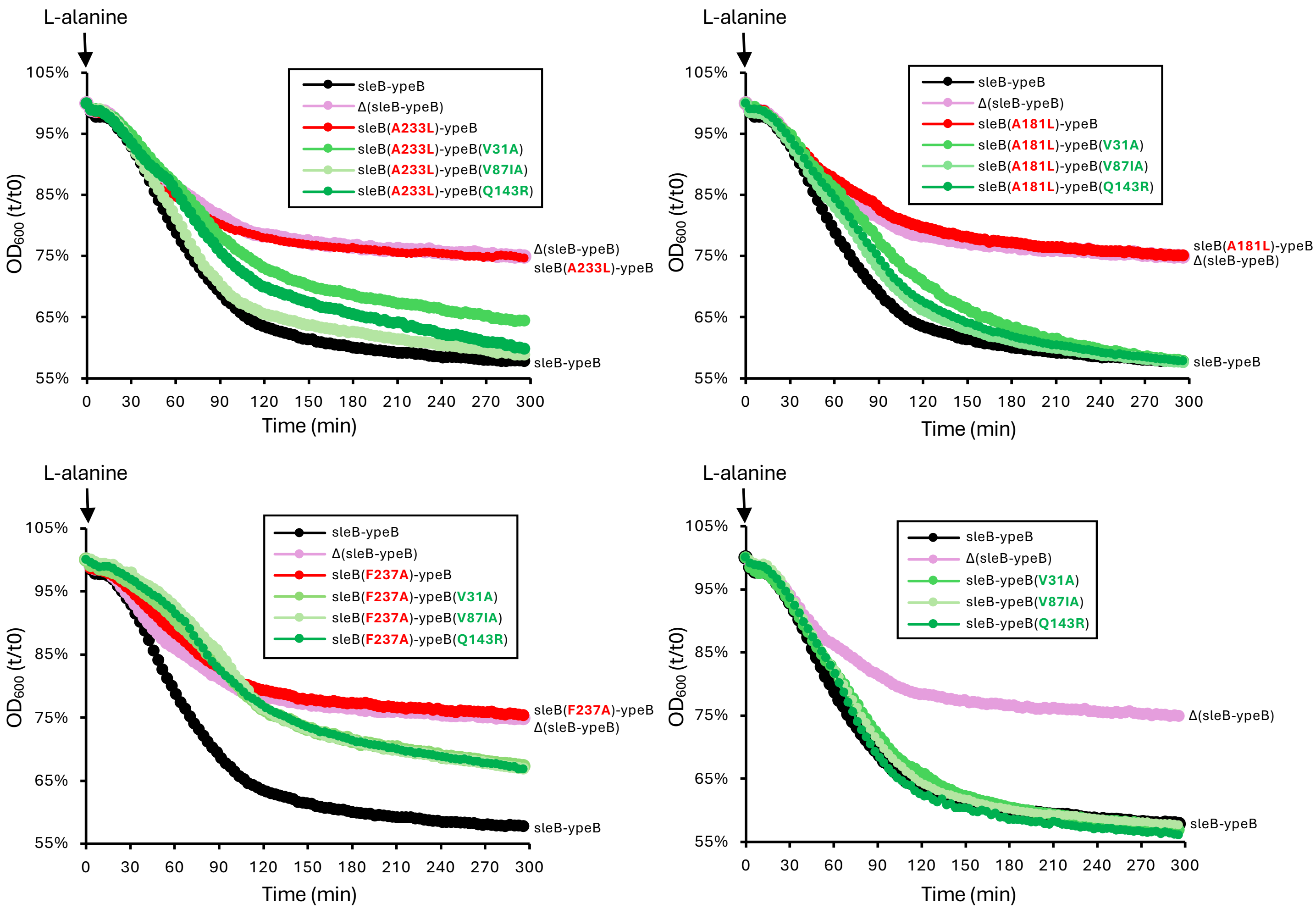


**Figure S4. YpeB suppressors mutants are germination-competent.** Representative graphs of spore germination in response to L-alanine as assayed by a reduction in optical density ($OD_{600}$). Histodenz-purified phase-bright spores of the indicated strains lacking *cwlJ*, were induced to germinate with 10 mM L-alanine, and $OD_{600}$ was monitored over time. The data are plotted as the percent reduction in $OD_{600}$ relative to time 0.

Figure S5
*Gao et al.*

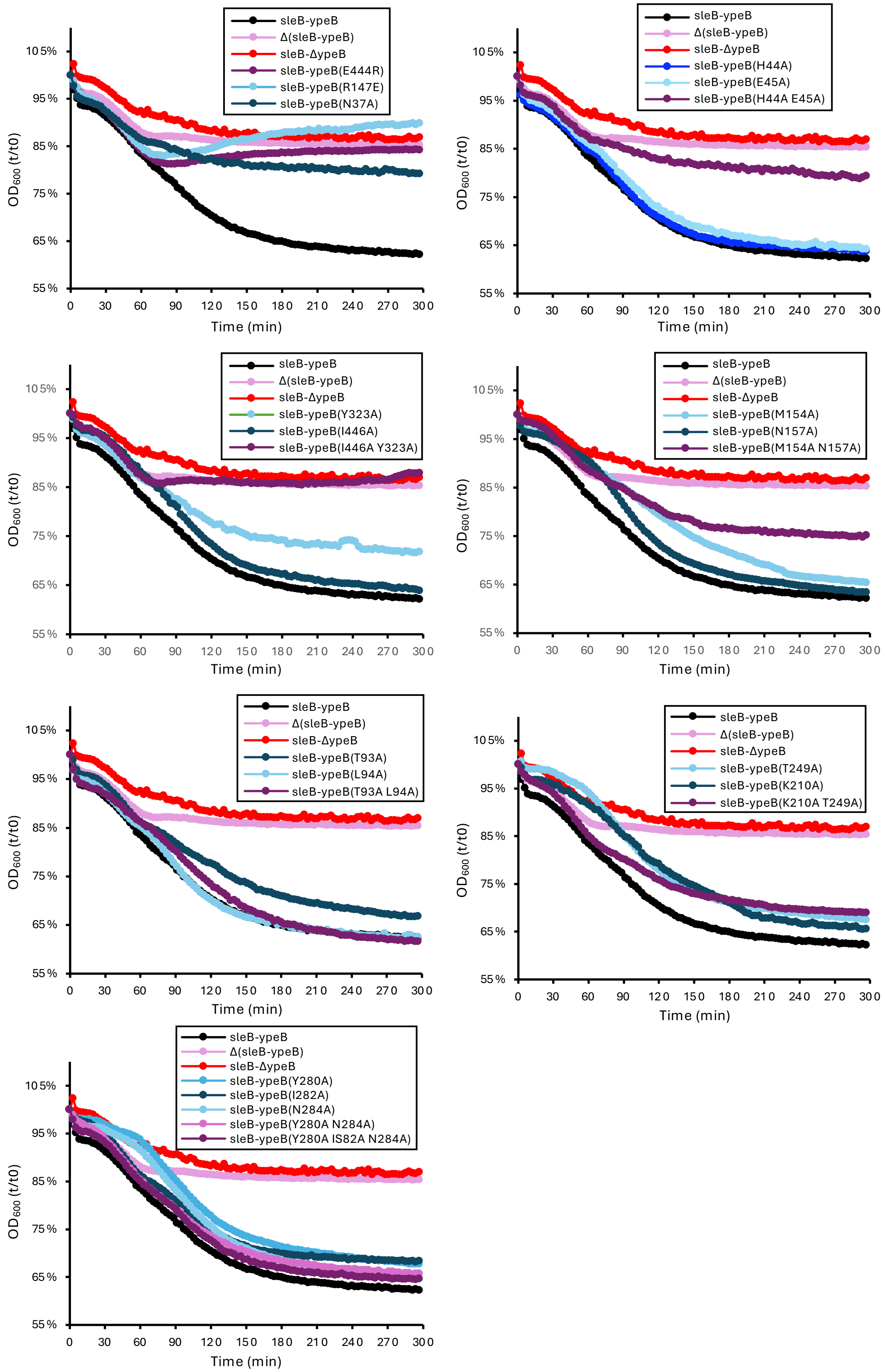


**Figure S5. Analysis of spore germination of the YpeB mutants.** Representative graphs of spore germination in response to L-alanine as assayed by a reduction in optical density ($OD_{600}$). Histodenz-purified phase-bright spores of the indicated strains lacking *cwlJ*, were induced to germinate with 10 mM L-alanine, and the $OD_{600}$ was monitored over time. The data are plotted as the percent reduction in $OD_{600}$ relative to time 0. In two cases, *ypeB*(M154A, N157A) and *ypeB*(K210A, T249A), the germination defects were more modest than expected based on the sporulation efficiencies in Figure 3B. Spores used for germination were generated on sporulation agar plates at 30˚C while the sporulation efficiency was determined from cells sporulated in liquid sporulation medium at 37˚C, potentially explaining the discrepancies.

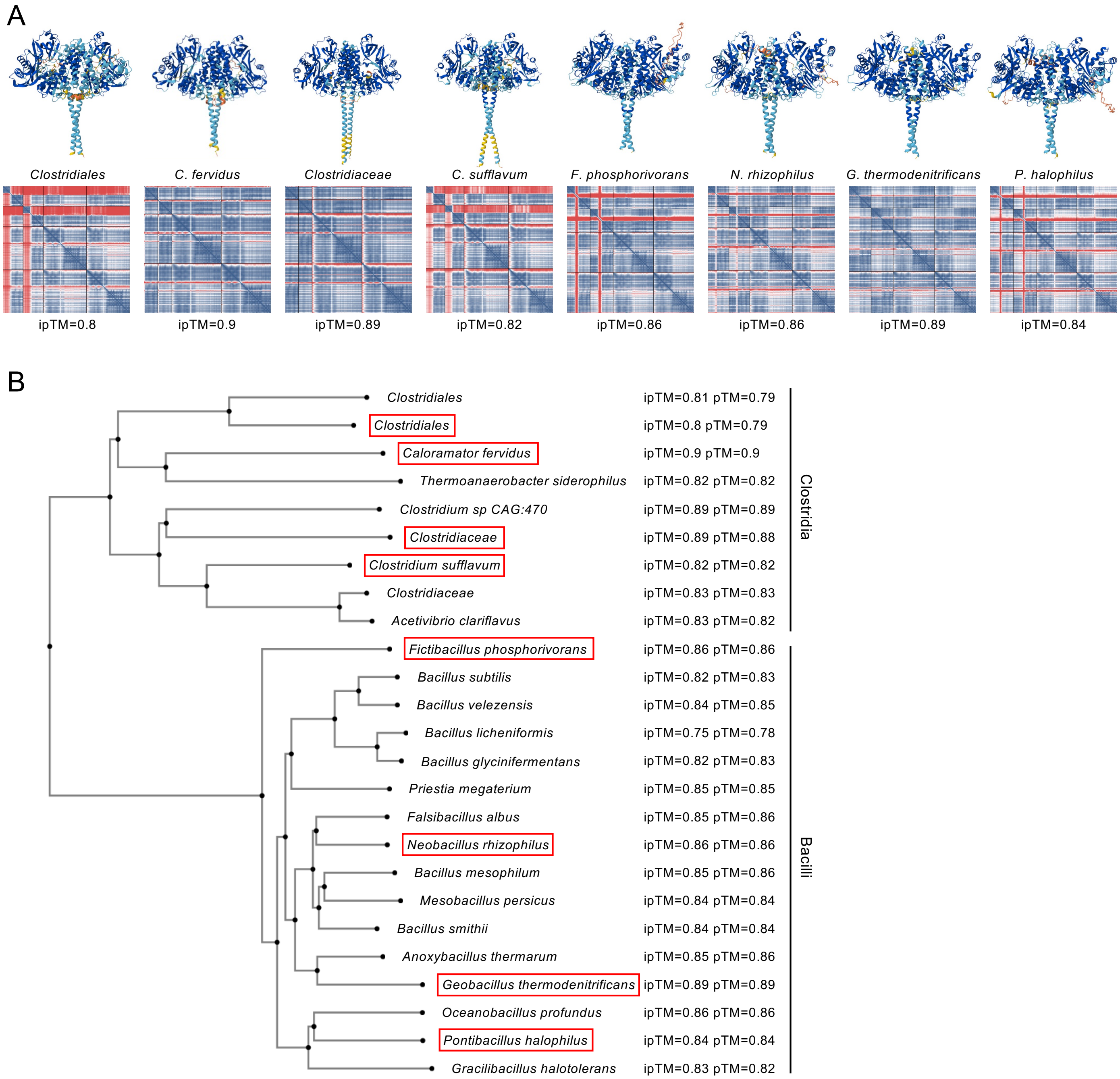


**Figure S6. The structure of the *B. subtilis* YpeB$_2$-SleB$_2$ complex is likely conserved across diverse endospore formers in the Bacillota. (A)** AlphaFold3-predicted structures of YpeB$_2$-SleB$_2$ tetrameric complexes from 8 endospore forming bacteria. The models are colored based based on the predicted local distance difference test (pLDDT), a per-residue measure of local confidence. The higher confidence scores are dark blue. Plots of the predicted alignment error (pAE) in Å of all residues against all residues for the top-ranked models are shown below. Low error (blue) corresponds to well-defined relative domain positions. The interface predicted template modeling score (ipTM) for each model is shown below the pAE plot. **(B)** Phylogenetic tree of spore-forming Clostridia and Bacilli for which a YpeB$_2$-SleB$_2$ tetrameric complex was predicted by AlphaFold3. The ipTM and template modeling (pTM) score for the top model is indicated to the right of the species. Uniprot IDs for all proteins analyzed can be found in Table S4. The predicted structures and pAE plots of the YpeB$_2$-SleB$_2$ complexes for the species boxed in red are shown in (A).

Figure S7
*Gao et al.*

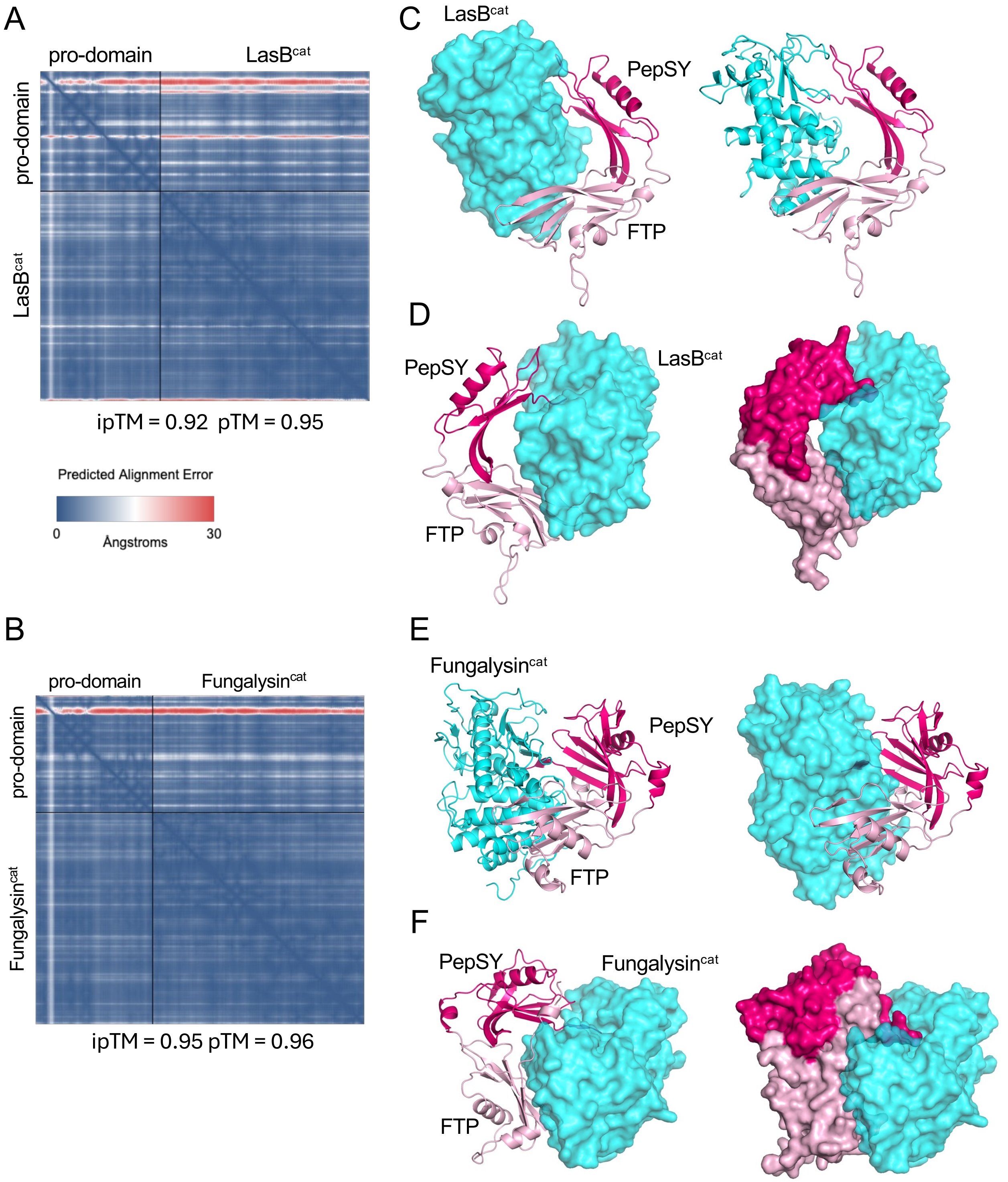


**Figure S7. LasB and Fungalysin pro-domains composed of PepSY and FTP domains embrace their catalytic domains. (A-B)** Plots of the predicted alignment error (pAE) in Å of the AlphaFold3 model of LasB and Fungalysin and their cleaved pro-domains. **(C)** Predicted structure of LasB's catalytic domain (LasB$^{cat}$) and its cleaved pro-domain. The PepSY domain (dark pink), FTP domain (light pink), and LasB$^{cat}$ (cyan) are rendered as ribbons and surface. **(D)** A rotated view of the predicted complex highlighting the "embrace" of the catalytic domain by both PepSY and FTP domains. **(E)** Predicted structure of Fugalysin's catalytic domain (Fungalysin$^{cat}$) and its cleaved pro-domain. The PepSY domain (dark pink), FTP domain (light pink), and Fungalysin$^{cat}$ (cyan) are rendered as ribbons and surface. **(D)** A rotated view of the predicted complex highlighting the "embrace" of the catalytic domain by both PepSY and FTP domains.

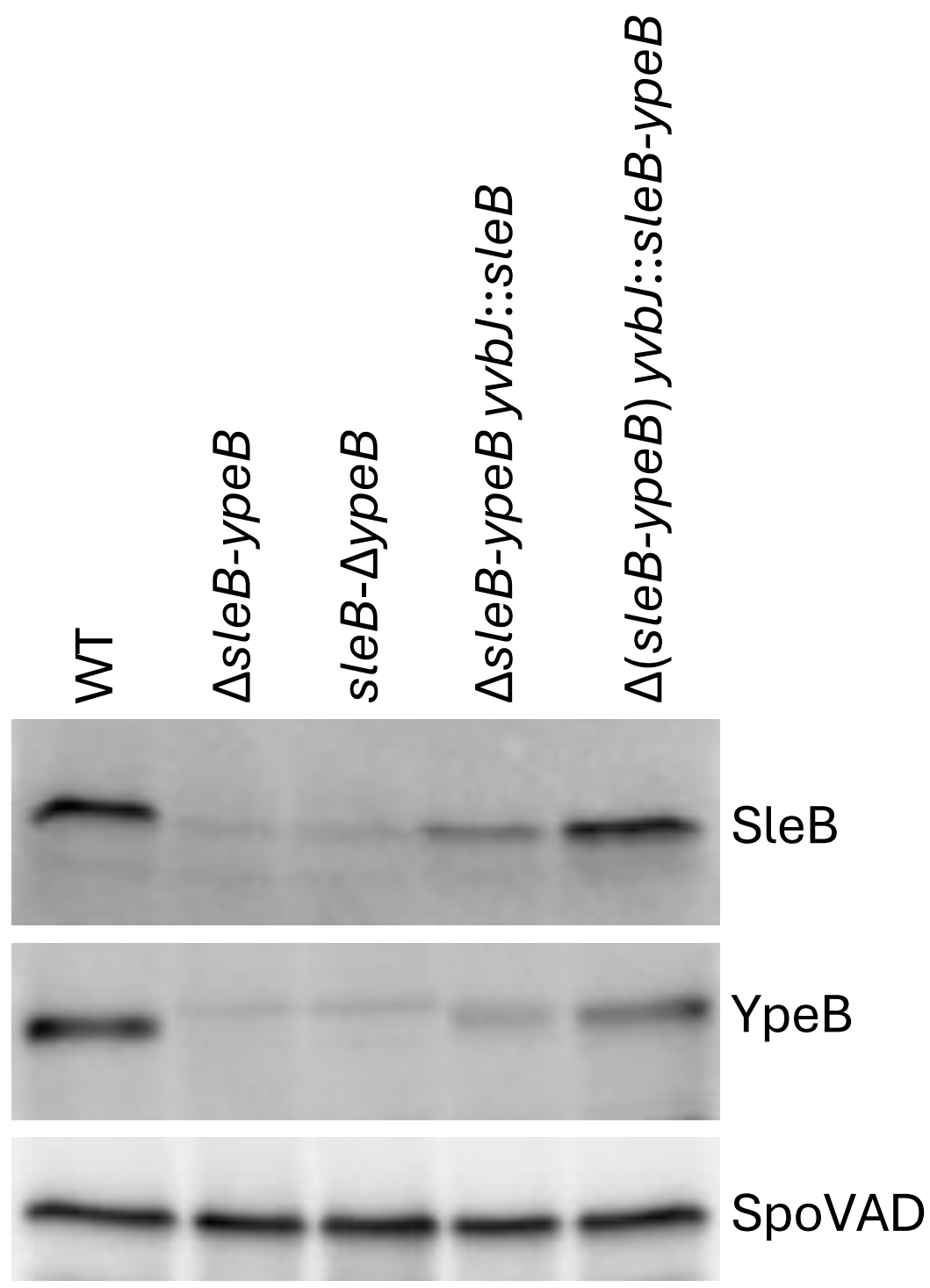


**Figure S8. The levels of SleB and YpeB in dormant spores are influenced by the presence of the two genes in a single operon.** Representative immunoblots of SleB and YpeB from spore lysates of the indicated strains. SpoVAD controls for loading.

**Supplemental Table 1. Strains used in this study**

| Strains | Genotype | Source | Figures |
|---|---|---|---|
| bDR2414 | *Bacillus subtilis 168 (trpC2) "wild-type"* | (2) | 1, S1 |
| bdr4178 | *ΔypeB::erm* | This work | S8 |
| bYG02 | *ΔsleB::lox72* | This work | S8 |
| bYG1073 | *ΔcwlJ::erm Δ(sleB-ypeB)::lox72 yvbJ::sleB(A181L)-ypeB(E101K) spec* | This work | 2 |
| bYG1074 | *ΔcwlJ::erm Δ(sleB-ypeB)::lox72 yvbJ::sleB(A181L)-ypeB(V31A) spec* | This work | 2, S4 |
| bYG1075 | *ΔcwlJ::erm Δ(sleB-ypeB)::lox72 yvbJ::sleB(A181L)-ypeB(Q143R) spec* | This work | 2, S4 |
| bYG1076 | *ΔcwlJ::erm Δ(sleB-ypeB)::lox72 yvbJ::sleB(A233L)-ypeB(V31A) spec* | This work | 2, S4 |
| bYG1077 | *ΔcwlJ::erm Δ(sleB-ypeB)::lox72 yvbJ::sleB(A233L)-ypeB(H84R) spec* | This work | 2 |
| bYG1078 | *ΔcwlJ::erm Δ(sleB-ypeB)::lox72 yvbJ::sleB(A233L)-ypeB(V87I) spec* | This work | 2, S4 |
| bYG1079 | *ΔcwlJ::erm Δ(sleB-ypeB)::lox72 yvbJ::sleB(A233L)-ypeB(Q143R) spec* | This work | 2, S4 |
| bYG1081 | *ΔcwlJ::erm Δ(sleB-ypeB)::lox72 yvbJ::sleB(A233L)-ypeB(V185I) spec* | This work | 2 |
| bYG1086 | *ΔcwlJ::erm Δ(sleB-ypeB)::lox72 yvbJ::sleB-ypeB(Q143R) spec* | This work | S4 |
| bYG1087 | *ΔcwlJ::erm Δ(sleB-ypeB)::lox72 yvbJ::sleB-ypeB(V31A) spec* | This work | S4 |
| bYG1089 | *ΔcwlJ::erm Δ(sleB-ypeB)::lox72 yvbJ::sleB-ypeB(V87I) spec* | This work | S4 |
| bYG1091 | *ΔcwlJ::erm Δ(sleB-ypeB)::lox72 yvbJ::sleB(F237A)-ypeB(E101K) spec* | This work | 2 |
| bYG1092 | *ΔcwlJ::erm Δ(sleB-ypeB)::lox72 yvbJ::sleB(F237A)-ypeB(Q143R) spec* | This work | 2, S4 |
| bYG1093 | *ΔcwlJ::erm Δ(sleB-ypeB)::lox72 yvbJ::sleB(F237A)-ypeB(V31A) spec* | This work | 2, S4 |
| bYG1094 | *ΔcwlJ::erm Δ(sleB-ypeB)::lox72 yvbJ::sleB(F237A)-ypeB(H84R) spec* | This work | 2 |
| bYG1095 | *ΔcwlJ::erm Δ(sleB-ypeB)::lox72 yvbJ::sleB(F237A)-ypeB(V87I) spec* | This work | 2, S4 |
| bYG1109 | *ΔcwlJ::erm Δ(sleB-ypeB)::lox72 yvbJ::sleB(F237A)-ypeB(V185I) spec* | This work | 2 |
| bYG1110 | *ΔcwlJ::erm Δ(sleB-ypeB)::lox72 yvbJ::sleB(A181L)-ypeB(V87I) spec* | This work | 2, S4 |
| bYG1111 | *ΔcwlJ::erm Δ(sleB-ypeB)::lox72 yvbJ::sleB(A181L)-ypeB(V185I) spec* | This work | 2 |
| bYG1112 | *ΔcwlJ::erm Δ(sleB-ypeB)::lox72 yvbJ::sleB(A233L)-ypeB(E101K) spec* | This work | 2 |
| bYG153 | *ΔsleB::lox72 yvbJ::sleB Cm* | This work | S8 |
| bYG159 | *Δ(sleB-ypeB)::lox72 yvbJ::sleB-ypeB cat* | This work | S8 |
| bYG1590 | *Δ(sleB-ypeB)::erm yvbJ::Pxyl-mYpet-ypeB cat* | This work | S2 |
| bYG1619 | *Δ(sleB-ypeB)::erm yvbJ::Pxyl-mYpet-ypeB cat amyE::Phyperspank-sleB spec* | This work | S2 |
| bYG1700 | *ΔcwlJ::erm Δ(sleB-ypeB)::lox72 yhdG::kan* | This work | 3, 4, S5 |
| bYG1704 | *ΔcwlJ::erm Δ(sleB-ypeB)::lox72 yhdG::sleB-ypeB(H44A E45A) spec* | This work | 3, S5 |
| bYG1707 | *ΔcwlJ::erm Δ(sleB-ypeB)::lox72 yhdG::sleB-ypeB(R147E) spec* | This work | 3, 4, S5 |
| bYG1708 | *ΔcwlJ::erm Δ(sleB-ypeB)::lox72 yhdG::sleB-ypeB(M154A N157A) spec* | This work | 3, S5 |
| bYG1710 | *ΔcwlJ::erm Δ(sleB-ypeB)::lox72 yhdG::sleB-ypeB(Y280A N284A) spec* | This work | 3, S5 |
| bYG1714 | *ΔcwlJ::erm Δ(sleB-ypeB)::lox72 yvbJ::sleB-ypeB spec* | This work | 2, 3, 4, S4, S5 |
| bYG1864 | *ΔcwlJ::lox72* | This work | 2, 4 |
| bYG1977 | *ΔcwlJ::erm Δ(sleB-ypeB)::lox72 yvbJ::sleB(R194A Q198A)-ypeB Cm* | This work | 2 |
| bYG1978 | *ΔcwlJ::erm Δ(sleB-ypeB)::lox72 yvbJ::Kan* | This work | 2, S4 |
| bYG2000 | *ΔcwlJ::erm Δ(sleB-ypeB)::lox72 yvbJ::sleB(A181V)-ypeB spec* | This work | 2 |
| bYG2001 | *ΔcwlJ::erm Δ(sleB-ypeB)::lox72 yvbJ::sleB(A233V)-ypeB spec* | This work | 2 |
| bYG2002 | *ΔcwlJ::erm Δ(sleB-ypeB)::lox72 yvbJ::sleB(Y251A)-ypeB spec* | This work | 2 |
| bYG2003 | *ΔcwlJ::erm Δ(sleB-ypeB)::lox72 yvbJ::sleB(N190A N191A)-ypeB spec* | This work | 2 |
| bYG2006 | *ΔcwlJ::erm Δ(sleB-ypeB)::lox72 yvbJ::sleB(N191A)-ypeB Cm* | This work | 2 |
| bYG2045 | *ΔcwlJ::erm Δ(sleB-ypeB)::lox72 yvbJ::sleB(F237A)-ypeB spec* | This work | 2, S4 |
| bYG2046 | *ΔcwlJ::erm Δ(sleB-ypeB)::lox72 yvbJ::sleB(A181L)-ypeB spec* | This work | 2, S4 |
| bYG2047 | *ΔcwlJ::erm Δ(sleB-ypeB)::lox72 yvbJ::sleB(A233L)-ypeB spec* | This work | 2, S4 |
| bYG2070 | *ΔcwlJ::erm Δ(sleB-ypeB)::lox72 yvbJ::sleB(N190A)-ypeB Cm* | This work | 2 |
| bYG2182 | *ΔcwlJ::erm Δ(sleB-ypeB)::lox72 yhdG::sleB-ypeB(N37A) spec* | This work | 3, S5 |
| bYG2184 | *ΔcwlJ::erm Δ(sleB-ypeB)::lox72 yhdG::sleB-ypeB(H44A) spec* | This work | 3, S5 |
| bYG2185 | *ΔcwlJ::erm Δ(sleB-ypeB)::lox72 yhdG::sleB-ypeB(E45A) spec* | This work | 3, S5 |

| | | | |
|---|---|---|---|
| bYG2187 | *ΔcwlJ::erm Δ(sleB-ypeB)::lox72 yhdG::sleB-ypeB(I282A) spec* | This work | 3, S5 |
| bYG2188 | *ΔcwlJ::erm Δ(sleB-ypeB)::lox72 yhdG::sleB-ypeB(T93A L94A) spec* | This work | 3, S5 |
| bYG2189 | *ΔcwlJ::erm Δ(sleB-ypeB)::lox72 yhdG::sleB-ypeB(I446A) spec* | This work | 3, S5 |
| bYG2190 | *ΔcwlJ::erm Δ(sleB-ypeB)::lox72 yhdG::sleB-ypeB(T249A) spec* | This work | 3, S5 |
| bYG2192 | *ΔcwlJ::erm Δ(sleB-ypeB)::lox72 yhdG::sleB-ypeB(Y280A I282A N284A) spec* | This work | 3, S5 |
| bYG2201 | *ΔcwlJ::erm Δ(sleB-ypeB)::lox72 yhdG::sleB-ypeB(Y323A) spec* | This work | 3, S5 |
| bYG2207 | *ΔcwlJ::erm Δ(sleB-ypeB)::lox72 yhdG::sleB-ypeB(I446A Y323A) spec* | This work | 3, S5 |
| bYG2208 | *ΔcwlJ::erm Δ(sleB-ypeB)::lox72 yhdG::sleB-ypeB(T249A K210A) spec* | This work | 3, S5 |
| bYG2252 | *ΔcwlJ::erm Δ(sleB-ypeB)::lox72 yhdG::sleB-ypeB(K210A) spec* | This work | 3, S5 |
| bYG2253 | *ΔcwlJ::erm Δ(sleB-ypeB)::lox72 yhdG::sleB-ypeB(Y280A) spec* | This work | 3, S5 |
| bYG2254 | *ΔcwlJ::erm Δ(sleB-ypeB)::lox72 yhdG::sleB-ypeB(N284A) spec* | This work | 3, S5 |
| bYG2256 | *ΔcwlJ::erm Δ(sleB-ypeB)::lox72 yhdG::sleB-ypeB(E444R) spec* | This work | 3, 4, S5 |
| bYG2258 | *ΔcwlJ::erm Δ(sleB-ypeB)::lox72 yhdG::sleB-ypeB(M154A) spec* | This work | 3, S5 |
| bYG2259 | *ΔcwlJ::erm Δ(sleB-ypeB)::lox72 yhdG::sleB-ypeB(N157A) spec* | This work | 3, S5 |
| bYG2260 | *ΔcwlJ::erm Δ(sleB-ypeB)::lox72 yhdG::sleB-ypeB(T93A) spec* | This work | 3, S5 |
| bYG2261 | *ΔcwlJ::erm Δ(sleB-ypeB)::lox72 yhdG::sleB-ypeB(L94A) spec* | This work | 3, S5 |
| bYG2327 | *ΔcwlJ::erm Δ(sleB-ypeB)::lox72 yhdG::sleB(N267I)-ypeB(E444R) spec* | This work | 4 |
| bYG2348 | *ΔcwlJ::erm Δ(sleB-ypeB)::lox72 yhdG::sleB(D192V)-ypeB(R147E) spec* | This work | 4 |
| bYG2377 | *ΔcwlJ::erm Δ(sleB-ypeB)::lox72 yhdG::sleB(N267I)-ypeB(E45K) spec* | This work | 4 |
| bYG2378 | *ΔcwlJ::erm Δ(sleB-ypeB)::lox72 yhdG::sleB(D192V)-ypeB(E45K) spec* | This work | 4 |
| bYG2381 | *ΔcwlJ::erm Δ(sleB-ypeB)::lox72 yhdG::sleB(N267I)-ypeB(R147E) spec* | This work | 4 |
| bYG2386 | *ΔcwlJ::lox72 ΔypeB::tet* | This work | 3, 4, S5 |
| bYG2405 | *ΔcwlJ::erm Δ(sleB-ypeB)::lox72 yhdG::sleB(D192V)-ypeB(M154A N157A) spec* | This work | 4, |
| bYG2411 | *ΔcwlJ::erm Δ(sleB-ypeB)::lox72 yhdG::sleB(D192V)-ypeB(E444R) spec* | This work | 4 |
| bYG2412 | *ΔcwlJ::erm Δ(sleB-ypeB)::lox72 yhdG::sleB(N267I)-ypeB spec* | This work | 4 |
| bYG2414 | *ΔcwlJ::erm Δ(sleB-ypeB)::lox72 yhdG::sleB(N267I)-ypeB(M154A N157A) spec* | This work | 4 |
| bYG2415 | *ΔcwlJ::erm Δ(sleB-ypeB)::lox72 yhdG::sleB(D192V)-ypeB spec* | This work | 4 |
| bYG64 | *ΔcwlJ::lox72 ΔsleB::lox72* | This work | 2, 4 |

**Supplemental Table 2. Plasmids used in this study.**

| Plasmid | Genotype | Source |
|---|---|---|
| pLA73 | *MCS-proC His-SUMO-FLAG-MCS (amp)* | (1) |
| pAM174 | *arabinose-inducible Ulp1[L403-K621] protease (cat)* | (3) |
| pCB43 | *yvbJ::spec (amp)* | laboratory stock |
| pCB06 | *yvbJ::cat (amp)* | laboratory stock |
| pCB33 | *yhdG::spec (amp)* | laboratory stock |
| pYG34 | *yvbJ::sleB (Cm)(amp)* | This work |
| pYG530 | *MCS-proC His-SUMO-FLAG-ypeB (amp)* | This work |
| pYG531 | *sleB His-SUMO-FLAG-ypeB (amp)* | This work |
| pYG532 | *MCS-proC His-SUMO-FLAG-ypeB(Bc) (amp)* | This work |
| pYG533 | *sleB(Bc) His-SUMO-FLAG-ypeB(Bc) (amp)* | This work |
| pYG547 | *SP(dsbA)-sleB(Bc) His-SUMO-FLAG-ypeB(Bc) (amp)* | This work |
| pYG548 | *SP(dsbA)-sleB(Bc) His-SUMO-FLAG-ypeB(Bc) (amp)* | This work |
| pYG549 | *SP(pelB)-sleB(Bc) His-SUMO-FLAG-ypeB(Bc) (amp)* | This work |
| pYG550 | *SP(pelB)-sleB His-SUMO-FLAG-ypeB (amp)* | This work |
| pYG566 | *SP(dsbA)-sleB-proC His-SUMO-FLAG-ypeB (amp)* | This work |
| pYG681 | *yvbJ::sleB-ypeB(spec)(amp)* | This work |
| pYG45 | *yvbJ::sleB-ypeB(cat)(amp)* | This work |
| pYG638 | *yvbJ::sleB N190A-ypeB(cat)(amp)* | This work |
| pYG636 | *yvbJ::sleB N191A-ypeB(cat)(amp)* | This work |
| pYG689 | *yvbJ::sleB N190A N190A-ypeB(spec)(amp)* | This work |
| pYG640 | *yvbJ::sleB R194A Q198A-ypeB(cat)(amp)* | This work |
| pYG683 | *yvbJ::sleB F237A-ypeB(spec)(amp)* | This work |
| pYG684 | *yvbJ::sleB A181L-ypeB(spec)(amp)* | This work |
| pYG685 | *yvbJ::sleB A233L-ypeB(spec)(amp)* | This work |
| pYG686 | *yvbJ::sleB A181V-ypeB(spec)(amp)* | This work |
| pYG687 | *yvbJ::sleB A233V-ypeB(spec)(amp)* | This work |
| pYG688 | *yvbJ::sleB Y251A-ypeB(spec)(amp)* | This work |
| pYG703 | *yvbJ::sleB A233L-ypeB E101K(spec)(amp)* | This work |
| pYG705 | *yvbJ::sleB A181L-ypeB V87I(spec)(amp)* | This work |
| pYG706 | *yvbJ::sleB A181L-ypeB V185I(spec)(amp)* | This work |
| pYG707 | *yvbJ::sleB F237A-ypeB E101K(spec)(amp)* | This work |
| pYG708 | *yvbJ::sleB F237A-ypeB Q143R(spec)(amp)* | This work |
| pYG709 | *yvbJ::sleB F237A-ypeB V31A(spec)(amp)* | This work |
| pYG710 | *yvbJ::sleB F237A-ypeB H84R(spec)(amp)* | This work |
| pYG711 | *yvbJ::sleB F237A-ypeB V87I(spec)(amp)* | This work |
| pYG712 | *yvbJ::sleB F237A-ypeB V185I(spec)(amp)* | This work |
| pYG535 | *yhdG::sleB-ypeB(spec)(amp)* | This work |
| pYG731 | *yhdG::sleB-ypeB H44A(spec)(amp)* | This work |
| pYG732 | *yhdG::sleB-ypeB E45A(spec)(amp)* | This work |
| pYG553 | *yhdG::sleB-ypeB H44A E45A(spec)(amp)* | This work |
| pYG556 | *yhdG::sleB-ypeB R147E(spec)(amp)* | This work |
| pYG757 | *yhdG::sleB-ypeB M154A(spec)(amp)* | This work |
| pYG758 | *yhdG::sleB-ypeB N157A(spec)(amp)* | This work |
| pYG557 | *yhdG::sleB-ypeB M154A N157A(spec)(amp)* | This work |
| pYG752 | *yhdG::sleB-ypeB Y280A(spec)(amp)* | This work |
| pYG753 | *yhdG::sleB-ypeB N284A(spec)(amp)* | This work |
| pYG559 | *yhdG::sleB-ypeB Y280A N284A(spec)(amp)* | This work |
| pYG736 | *yhdG::sleB-ypeB Y280A I282A N284A(spec)(amp)* | This work |

| | | |
|---|---|---|
| pYG733 | *yhdG::sleB-ypeB I282A(spec)(amp)* | This work |
| pYG734 | *yhdG::sleB-ypeB T249A(spec)(amp)* | This work |
| pYG751 | *yhdG::sleB-ypeB K210A(spec)(amp)* | This work |
| pYG742 | *yhdG::sleB-ypeB K210A T249A(spec)(amp)* | This work |
| pYG735 | *yhdG::sleB-ypeB Y323A(spec)(amp)* | This work |
| pYG739 | *yhdG::sleB-ypeB I446A(spec)(amp)* | This work |
| pYG740 | *yhdG::sleB-ypeB Y323A I446A(spec)(amp)* | This work |
| pYG759 | *yhdG::sleB-ypeB T93A(spec)(amp)* | This work |
| pYG760 | *yhdG::sleB-ypeB L94A(spec)(amp)* | This work |
| pYG741 | *yhdG::sleB-ypeB T93A L94A(spec)(amp)* | This work |
| pYG754 | *yhdG::sleB-ypeB E444A(spec)(amp)* | This work |
| pYG755 | *yhdG::sleB-ypeB E444R(spec)(amp)* | This work |
| pYG767 | *yhdG::*$P_{sleB}$*-(HindIII-XhoI)-ypeB R147E (amp)* | This work |
| pYG768 | *yhdG::PsleB-(HindIII-XhoI)-ypeB E444R (amp)* | This work |
| pYG779 | *yhdG::sleB N267I-ypeB R147E(spec)(amp)* | This work |
| pYG780 | *yhdG::sleB D192V-ypeB E444R(spec)(amp)* | This work |
| pYG783 | *yhdG::sleB N267I-ypeB E45K(spec)(amp)* | This work |
| pYG784 | *yhdG::sleB D192V-ypeB E45K(spec)(amp)* | This work |
| pYG796 | *yhdG::sleB D192V-ypeB M154A N157A(spec)(amp)* | This work |
| pYG799 | *yhdG::sleB N267I-ypeB M154A N157A(spec)(amp)* | This work |
| pYG802 | *yhdG::sleB D192V-ypeB(spec)(amp)* | This work |
| pYG803 | *yhdG::sleB N267I-ypeB(spec)(amp)* | This work |

**Supplemental Table 3. Oligonucleotides used in this study.**

| oligo | sequence | use |
|---|---|---|
| oCB63 | gactggtttttgctcagacaa | pYG767, pYG768 |
| oCB120 | cgcGAATTCGGTGGTTTGGTTTGCGTAAAGCG | pYG34 |
| oCB121 | cgcGGATCCTCTGATCATTTATCACACCTC | pYG34 |
| oYG1002 | ttgtctgagcaaaaaccagtc | pYG767, pYG768 |
| oYG1014 | gcggccgcataatgcttaagtcgaacag | pYG547, pYG548, pYG549, pYG550 |
| oYG1015 | GCTGGTTTAGTTTTAGCGTTTAGCGCATCGGCGGCAttttctaatcaagtcattcaaag | pYG547 |
| oYG1016 | atgAAAAAGATTTGGCTGGCGCTGGCTGGTTTAGTTTTAGCGTTTAG | pYG547, pYG548 |
| oYG1017 | ctttaagaaggagatataccatggatgAAAAAGATTTGGCTGGCGCT | pYG547, pYG548, pYG566 |
| oYG1018 | GCTGGTTTAGTTTTAGCGTTTAGCGCATCGGCGGCATTTTCGAATCAGGTCATTCAAAG | pYG548 |
| oYG1019 | CTGCTGCTCCTCGCTGCCCAGCCGGCGATGGCCGCAttttctaatcaagtcattcaaag | pYG549 |
| oYG1020 | atgAAATACCTGCTGCCGACCGCTGCTGCTGGTCTGCTGCTCCTCGCTGCCCAG | pYG549, pYG550 |
| oYG1021 | ctttaagaaggagatataccatggatgAAATACCTGCTGCCGACCGCT | pYG549, pYG550 |
| oYG1022 | CTGCTGCTCCTCGCTGCCCAGCCGGCGATGGCCGCATTTTCGAATCAGGTCATTCAAAG | pYG550 |
| oYG1026 | GAAAATAACTATCAGCGGGCGTTTGCTGCGCTTACCTATCAGGTGGATCAGCT | pYG553 |
| oYG1027 | AGCTGATCCACCTGATAGGTAAGCGCAGCAAACGCCCGCTGATAGTTATTTTC | pYG553 |
| oYG1032 | CAGTCCGAAGATATACAAAATGAATTGGAACATGTTCAGCACCTTGTCATGAG | pYG556 |
| oYG1033 | CTCATGACAAGGTGCTGAACATGTTCCAATTCATTTTGTATATCTTCGGACTG | pYG556 |
| oYG1034 | TGCGTCATGTTCAGCACCTTGTCGCGAGCAAAGCCCTTCGCTGGATGGACGTAGAAATG | pYG557 |
| oYG1035 | CATTTCTACGTCCATCCAGCGAAGGGCTTTGCTCGCGACAAGGTGCTGAACATGACGCA | pYG557 |
| oYG1038 | AGAAGGGCGGGCATCCGGTAGCCTTGATCCAAGCCAGAGAAGTGAAAGATCAGAAAATC | pYG559 |
| oYG1039 | GATTTTCTGATCTTTCACTTCTCTGGCTTGGATCAAGGCTACCGGATGCCCGCCCTTCT | pYG559 |
| oYG105 | ctggcGAATTCAAGTGGCTTACTCTTTCTTTAGTTG | pYG681, pYG45, pYG535 |
| oYG1050 | ctctggcggaagcggaggatccACTAGTGAAGACCAAGTAGATCCGCGGCTC | pYG566 |
| oYG1051 | ggatcctccgcttccgccagagcctccCTCACAGAAAATGTGTTTACCGATTC | pYG566 |
| oYG106 | cagcgGGATCCCACGTTTTTATTAAAGCACGTAATG | pYG681, pYG45, pYG535 |
| oYG1175 | CAAATTCAGTAGCGGGGGTTATTGCTGAGCCGCTTGCCTTCACAGCA | pYG683 |
| oYG1176 | TGCTGTGAAGGCAAGCGGCTCAGCAATAACCCCCGCTACTGAATTTG | pYG683 |
| oYG1177 | CAGCAGTAGCCGACGGACAAATTGCCATGCAGCCGAATGAAACGGCAC | pYG688 |
| oYG1178 | GTGCCGTTTCATTCGGCTGCATGGCAATTTGTCCGTCGGCTACTGCTG | pYG688 |
| oYG1179 | AGCCCGTTATTTCCAAATTCAGTACTGGGGGTTATTTTTGAGCCGCTTG | pYG685 |
| oYG1180 | CAAGCGGCTCAAAAATAACCCCCAGTACTGAATTTGGAAATAACGGGCT | pYG685 |
| oYG1181 | AGCCCGTTATTTCCAAATTCAGTAGTGGGGGTTATTTTTGAGCCGCTTG | pYG687 |
| oYG1182 | CAAGCGGCTCAAAAATAACCCCCACTACTGAATTTGGAAATAACGGGCT | pYG687 |
| oYG1183 | GAACATGCCTGGTGGATTTTCCAACGCCGATATCAGGCTGCTTGCTCAAG | pYG636 |
| oYG1184 | CTTGAGCAAGCAGCCTGATATCGGCGTTGGAAAATCCACCAGGCATGTTC | pYG636 |
| oYG1187 | AGCGAACATGCCTGGTGGATTTTCCGCCAACGATATCAGGCTGCTTGCTC | pYG638 |

| | | |
|---|---|---|
| oYG1188 | GAGCAAGCAGCCTGATATCGTTGGCGGAAAATCCACCAGGCATGTTCGCT | pYG638 |
| oYG1189 | GAACATGCCTGGTGGATTTTCCGCCGCCGATATCAGGCTGCTTGCTCAAG | pYG689 |
| oYG1190 | CTTGAGCAAGCAGCCTGATATCGGCGGCGGAAAATCCACCAGGCATGTTC | pYG689 |
| oYG1191 | TGGTGGATTTTCCAACAACGATATCGCGCTGCTTGCTGCAGCGGTTTATGGCGAAGCCC | pYG640 |
| oYG1192 | GGGCTTCGCCATAAACCGCTGCAGCAAGCAGCGCGATATCGTTGTTGGAAAATCCACCA | pYG640 |
| oYG1193 | AGCAAGAAAACAGGATGCGGTAGTAGCGAACATGCCTGGTGGATTTTC | pYG686 |
| oYG1194 | GAAAATCCACCAGGCATGTTCGCTACTACCGCATCCTGTTTTCTTGCT | pYG686 |
| oYG1195 | AGCAAGAAAACAGGATGCGGTACTAGCGAACATGCCTGGTGGATTTTC | pYG684 |
| oYG1196 | GAAAATCCACCAGGCATGTTCGCTAGTACCGCATCCTGTTTTCTTGCT | pYG684 |
| oYG1268 | ACATTAATGCCGTTTAATAAAACTAAAGAGCTATTATCAAAGATCGGCGA | pYG703, pYG707 |
| oYG1269 | TCGCCGATCTTTGATAATAGCTCTTTAGTTTTATTAAACGGCATTAATGT | pYG703, pYG707 |
| oYG1270 | CTATATCAGCAGTCCGAAGATATACGAAATGAATTGCGTCATGTTCAG | pYG708 |
| oYG1271 | CTGAACATGACGCAATTCATTTCGTATATCTTCGGACTGCTGATATAG | pYG708 |
| oYG1272 | GAACACCAGGAAAAAGACGCAGCTCTTCTTCATGCTGAAAATAACTATC | pYG709 |
| oYG1273 | GATAGTTATTTTCAGCATGAAGAAGAGCTGCGTCTTTTTCCTGGTGTTC | pYG709 |
| oYG1274 | GTGGAGGATTACATCAGAAGCTCGTAACAGCGTCAGTCAGCTGCCGCT | pYG710 |
| oYG1275 | AGCGGCAGCTGACTGACGCTGTTACGAGCTTCTGATGTAATCCTCCAC | pYG710 |
| oYG1276 | ACATCAGAAGCTCATAACAGCATCAGTCAGCTGCCGCTTACATTAATG | pYG705, pYG711 |
| oYG1277 | CATTAATGTAAGCGGCAGCTGACTGATGCTGTTATGAGCTTCTGATGT | pYG705, pYG711 |
| oYG1278 | ACGATTATCAACAGCTTTAAAACAATCGAAAAAAATGTTGGTGCATTC | pYG706, pYG712 |
| oYG1279 | GAATGCACCAACATTTTTTTCGATTGTTTTAAAGCTGTTGATAATCGT | pYG706, pYG712 |
| oYG1319 | GAAAATAACTATCAGCGGGCGTTTGCTGAGCTTACCTATCAGGTGGA | pYG731 |
| oYG1320 | TCCACCTGATAGGTAAGCTCAGCAAACGCCCGCTGATAGTTATTTTC | pYG731 |
| oYG1321 | ACTATCAGCGGGCGTTTCATGCGCTTACCTATCAGGTGGATCAGC | pYG732 |
| oYG1322 | GCTGATCCACCTGATAGGTAAGCGCATGAAACGCCCGCTGATAGT | pYG732 |
| oYG1323 | AGGGCGGGCATCCGGTATACTTGGCCCAAAACAGAGAAGTGAAAGATC | pYG733 |
| oYG1324 | GATCTTTCACTTCTCTGTTTTGGGCCAAGTATACCGGATGCCCGCCCT | pYG733 |
| oYG1325 | AAGTGGTAAAGAGCGGAAAAAAAGCAAATCGCGATGTATATAGCA | pYG734, pYG742 |
| oYG1326 | TGCTATATACATCGCGATTTGCTTTTTTTCCGCTCTTTACCACTT | pYG734, pYG742 |
| oYG1327 | GGAAATTGATGAAAGTGCCCAAGCTGATAAAATCGGTGTATTTTCATATG | pYG735 |
| oYG1328 | CATATGAAAATACACCGATTTTATCAGCTTGGGCACTTTCATCAATTTCC | pYG735 |
| oYG1329 | AGGGCGGGCATCCGGTAGCCTTGGCCCAAGCCAGAGAAGTGAAAGATC | pYG736 |
| oYG1330 | GATCTTTCACTTCTCTGGCTTGGGCCAAGGCTACCGGATGCCCGCCCT | pYG736 |
| oYG1331 | GAAAAACTAAAAAATGCAGAACCTGCATATAAAGACCTATAAAAACAGGAG | pYG739, pYG740 |
| oYG1332 | CTCCTGTTTTTATAGGTCTTTATATGCAGGTTCTGCATTTTTTAGTTTTTC | pYG739, pYG740 |
| oYG1333 | CAGCGTCAGTCAGCTGCCGCTTGCAGCAATGCCGTTTAATAAAACTGAAGA | pYG741 |
| oYG1334 | TCTTCAGTTTTATTAAACGGCATTGCTGCAAGCGGCAGCTGACTGACGCTG | pYG741 |
| oYG1335 | CACCAGTACGAAAAAAGAAGAGGCAGGCTTCAGCCATCTGAAGGGA | pYG751 |
| oYG1336 | TCCCTTCAGATGGCTGAAGCCTGCCTCTTCTTTTTTCGTACTGGTG | pYG751 |

| | | |
|---|---|---|
| oYG1350 | AGAAGGGCGGCATCCGGTAGCCTTGATCCAAAACAGAGAAGT | pYG752 |
| oYG1351 | ACTTCTCTGTTTTGGATCAAGGCTACCGGATGCCCGCCCTTCT | pYG752 |
| oYG1352 | CATCCGGTATACTTGATCCAAGCCAGAGAAGTGAAAGATCAGAAAATC | pYG753 |
| oYG1353 | GATTTTCTGATCTTTCACTTCTCTGGCTTGGATCAAGTATACCGGATG | pYG753 |
| oYG1356 | GTTGAAAAACTAAAAAATGCACGACCTATATATAAAGACCTATAAAAAC | pYG755 |
| oYG1357 | GTTTTTATAGGTCTTTATATATAGGTCGTGCATTTTTTAGTTTTTCAAC | pYG755 |
| oYG1360 | GTCATGTTCAGCACCTTGTCGCGAGCAAAAACCTTCGCTGGA | pYG757 |
| oYG1361 | TCCAGCGAAGGTTTTTGCTCGCGACAAGGTGCTGAACATGAC | pYG757 |
| oYG1362 | AGCACCTTGTCATGAGCAAAGCCCTTCGCTGGATGGACGTAGA | pYG758 |
| oYG1363 | TCTACGTCCATCCAGCGAAGGGCTTTGCTCATGACAAGGTGCT | pYG758 |
| oYG1364 | AGCGTCAGTCAGCTGCCGCTTGCATTAATGCCGTTTAATAAAAC | pYG759 |
| oYG1365 | GTTTTATTAAACGGCATTAATGCAAGCGGCAGCTGACTGACGCT | pYG759 |
| oYG1366 | GTCAGTCAGCTGCCGCTTACAGCAATGCCGTTTAATAAAACTGA | pYG760 |
| oYG1367 | TCAGTTTTATTAAACGGCATTGCTGTAAGCGGCAGCTGACTGAC | pYG760 |
| oYG1407 | AGCAGTGCTGGATGCCATCATTGGCTGGGACCCATCAGAGGAAG | pYG779, pYG783, pYG799, pYG803 |
| oYG1408 | CTTCCTCTGATGGGTCCCAGCCAATGATGGCATCCAGCACTGCT | pYG779, pYG783, pYG799, pYG803 |
| oYG1409 | CTGGTGGATTTTCCAACAACGTTATCAGGCTGCTTGCTCAAGC | pYG780, pYG784, pYG796, pYG802 |
| oYG1410 | GCTTGAGCAAGCAGCCTGATAACGTTGTTGGAAAATCCACCAG | pYG780, pYG784, pYG796, pYG802 |
| oYG190 | CATCCATGgtatatctccttcttaaagttaaac | pYG547, pYG548, pYG549, pYG550, pYG556 |
| oYG992 | GGTGTTGTTTCAAGGCCCTGGGgGATCCATCAGAGGAATTTTAATCGCCGTGCTTG | pYG530 |
| oYG993 | gcagcggtttctttaccagacTCGAGTTATAGGTCTTTATATATAGGTTCTGCA | pYG530 |
| oYG994 | ttgtttaactttaagaaggagatataccatggATGAAGTCCAAAGGATCGATTATGGCA | pYG531 |
| oYG994-F | GGTGTTGTTTCAAGGCCCTGGGgGATCCttacgaggtattatcattgtattattaac | pYG532 |
| oYG995 | ctgttcgacttaagcattatgcggccgcCTACTCACAGAAAATGTGTTTACCGATTC | pYG531, pYG548, pYG550 |
| oYG995-R | gcagcggtttctttaccagacTCGAGttaatcataaattttttcaacagcttgca | pYG532 |
| oYG996 | tgtttaactttaagaaggagatataccatggatgcgccaaaaagctatttttaaaatag | pYG533 |
| oYG997 | ctgttcgacttaagcattatgcggccgcctatttacagaaaatatgtttaccga | pYG533, pYG547, pYG549 |
| oYG998 | AGAAGCTTatACTAGTgtCTCGAGATATGAGAAAGCATAAAAAGAGGTGTGA | pYG767, pYG768 |
| oYG999 | CTCGAGacACTAGTatAAGCTTCTTTTCAAGCCTCCTACTGCAAATTTTTAAG | pYG767, pYG768 |

**Table S4**
**Proteins analyzed in Figure S6**

| | | |
|---|---|---|
| *Bacillus licheniformis* (strain ATCC 14580) | SleB: Q65I05_BACLD | YpeB: Q65I06_BACLD |
| *Falsibacillus albus* | SleB: A0A3L7JW42_9BACI | YpeB: A0A3L7JW36_9BACI |
| *Anoxybacillus thermarum* | SleB: A0A0D0S0X8_9BACL | YpeB: A0A0D0RSH9_9BACL |
| *Priestia megaterium* (strain ATCC 14581) | SleB: A0A0B6ABB4_PRIM2 | YpeB: A0A0B6AYZ4_PRIM2 |
| *Geobacillus thermodenitrificans* (strain NG80-2) | SleB: A4IQB2_GEOTN | YpeB: A4IQB1_GEOTN |
| *Bacillus glycinifermentans* | SleB: A0A0T6BTH1_9BACI | YpeB: A0A0T6BTG2_9BACI |
| *Fictibacillus phosphorivorans* | SleB: A0A160IMZ9_9BACL | YpeB: A0A160IPR8_9BACL |
| *Oceanobacillus profundus* | SleB: A0A417YFT7_9BACI | YpeB: A0A417YFT1_9BACI |
| *Neobacillus rhizophilus* | SleB: A0A942UAT4_9BACI | YpeB: A0A942UBT1_9BACI |
| *Pontibacillus halophilus* (JSM 076056) | SleB: A0A0A5GJL4_9BACI | YpeB: A0A0A5GLS1_9BACI |
| *Mesobacillus persicus* | SleB: A0A1H8BSK3_9BACI | YpeB: A0A1H8BSK2_9BACI |
| *Bacillus mesophilum* | SleB: A0A7V7RMU9_9BACI | YpeB: A0A7V7UYQ1_9BACI |
| *Gracilibacillus halotolerans* | SleB: A0A841RGC4_9BACI | YpeB: A0A841RCJ7_9BACI |
| *Bacillus smithii* (7_3_47FAA) | SleB: N0AXJ5 | YpeB: N0B254 |
| *Clostridiales bacterium* | SleB: A0A972A6X9 | YpeB: A0A971VA40 |
| *Clostridium sp.* (CAG:470) | SleB: R7FH44 | YpeB: R7FHP6 |
| *Caloramator fervidus* | SleB: A0A1H5RLY6_9CLOT | YpeB: A0A1H5RK67_9CLOT |
| *Thermoanaerobacter siderophilus* SR4 | SleB: I8QYE3_9THEO | YpeB: I9ADH5_9THEO |
| *Clostridiales bacterium* | SleB: A0A971ZAI8 | YpeB: A0A971ZAP5 |
| *Clostridiaceae bacterium* | SleB: A0A970JCK8 | YpeB: A0A970JCX8 |
| *Clostridium sufflavum* (DSM 19573) | SleB: A0A318XME9_9FIRM | YpeB: A0A318XPE2_9FIRM |
| *Clostridiaceae bacterium* | SleB: A0A7X8JVJ4 | YpeB: A0A7X8JVG8 |
| *Acetivibrio clariflavus* (strain DSM 19732) | SleB: G8LZE9_ACECE | YpeB: G8LZF0_ACECE |
| *Bacillus velezensis* (strain DSM 23117) | SleB: A7Z643_BACVZ | YpeB: A7Z642_BACVZ |

**SUPPLEMENTAL REFERENCES**


1. Gao Y, Barajas-Ornelas RDC, Amon JD, Ramírez-Guadiana FH, Alon A, Brock KP, Marks DS, Kruse AC, Rudner DZ. 2022. The SpoVA membrane complex is required for dipicolinic acid import during sporulation and export during germination. Genes Dev 36:634-646.

2. Zeigler DR, Prágai Z, Rodriguez S, Chevreux B, Muffler A, Albert T, Bai R, Wyss M, Perkins JB. 2008. The origins of 168, W23, and other *Bacillus subtilis* legacy strains. J Bacteriol 190:6983-95.

3. Meeske AJ, Riley EP, Robins WP, Uehara T, Mekalanos JJ, Kahne D, Walker S, Kruse AC, Bernhardt TG, Rudner DZ. 2016. SEDS proteins are a widespread family of bacterial cell wall polymerases. Nature 537:634-638.
